# Replication of Strategic and Interactive Writing Instruction in a Nationwide Randomized Controlled Trial

**DOI:** 10.3390/bs16010086

**Published:** 2026-01-07

**Authors:** Kimberly Wolbers, Hannah M. Dostal, Lee Branum-Martin, Steve Graham, Jennifer Renée Kilpatrick, Thomas Allen, Rachel Saulsburry, Leala Holcomb, Kelsey Spurgin

**Affiliations:** 1 Theory and Practice in Teacher Education, The University of Tennessee, Knoxville, TN 37996, USA; 2 Department of Curriculum and Instruction, University of Connecticut, Storrs, CT 06269, USA; hannah.dostal@uconn.edu; 3 Department of Psychology, Georgia State University, Atlanta, GA 30302, USA; 4 Mary Lou Fulton College for Teaching and Learning Innovation, Arizona State University, Tempe, AZ 85287, USA; steve.graham@asu.edu; 5 Teaching, Learning, and Curriculum, University of North Florida, Jacksonville, FL 32224, USA; jrkilpatrick@unf.edu; 6 Gallaudet University, Washington, DC 20002, USA; 7 Aiken County Public School District, Aiken, SC 29803, USA; 8 Department of Special Education, Ball State University, Muncie, IN 47306, USA

**Keywords:** deaf, writing instruction, elementary, randomized controlled trial, efficacy study, strategic, interactive, metalinguistic

## Abstract

This study reports findings from a nationwide replication and the second randomized controlled trial (RCT) of Strategic and Interactive Writing Instruction (SIWI), a linguistically responsive framework for teaching writing to deaf students. A total of 50 teachers and their 294 students in grades 3–6 were randomly assigned to either SIWI or business-as-usual (BAU) instruction. Writing outcomes were assessed with trait-based rubrics and the Structured Analysis of Written Language (SAWL) in two genres (recount and information report), along with the Woodcock–Johnson IV broad written language composite and genre-specific motivation surveys administered at the beginning and end of the school year. Students receiving SIWI outperformed peers in the BAU group on writing traits across both genres, with effect sizes ranging from moderately large (d = 0.70) for informational reports to very large (d = 1.11) for recounts. On the SAWL, SIWI students demonstrated significantly greater gains in grammatical clarity on recount writing, as measured by the word efficiency ratio, with a moderate effect size (d = 0.64), although this effect was not observed for information reports. Students in the treatment group also reported significantly higher motivation for both genres. Unlike the prior RCT, no statistically significant differences emerged on the broad written language measure (d = 0.27). This may reflect spurious findings in the previous study or limitations in this study caused by the COVID-19 pandemic. Nonetheless, the effect size observed suggests some practical importance that warrants further investigation. Findings provide robust evidence that SIWI positively impacts deaf elementary students’ writing development and motivation, particularly for recount genres, while underscoring the importance of replication for understanding the generalizability of intervention effects.

## 1. Literature Review

Deaf and hard-of-hearing learners (hereafter, referred to as deaf) exhibit a wide range of writing abilities, reflecting the diversity of their language experiences and access to quality instruction. Writing development among deaf learners is deeply influenced by their individual linguistic backgrounds. This may include limited access to spoken English, exposure to a signed language, and/or experiences of language deprivation (Holcomb et al., 2023a). As Beal et al. (2024) and Hall and De Anda (2021) note, the heterogeneity in language proficiency and modalities among deaf children forms the foundation for their literacy development and academic engagement. Additionally, translanguaging practices are frequently observed in the writing of deaf students who communicate through multiple languages and modalities. Rather than fully separating their expressions by English and American Sign Language (ASL), these students draw on their unified linguistic system during meaning-making processes. These diverse patterns in writing development differ from those typically observed among hearing peers and highlight the need for specialized, linguistically responsive writing instruction that validates and expands students’ full linguistic repertoires.

### 1.1. Writing Instruction and Deaf Students

Writing instruction for deaf students must take into account the linguistic diversity and language access needs described above. Unfortunately, research literature suggests that many teachers lack training and evidence-based curricula or frameworks for addressing these complex needs (Courson, 2022; Enns et al., 2007). Studies examining classroom instruction reveal that, while teachers of deaf students feel that writing instruction is important, skills are often taught with limited strategic or explicit attention to language development (H. M. Dostal et al., 2018). Teachers report relying on commercial curricula designed for general education or focusing on lower-level skills such as spelling and grammar rather than translating ideas or synthesizing information into cohesive, organized text (K. Wolbers et al., 2023a). Due to an absence of evidence-based guidance for writing instruction (Strassman & Schirmer, 2013; Williams & Mayer, 2015), writing opportunities for deaf students may be infrequent, removed from authentic purpose, or insufficiently scaffolded. Internationally, writing development among DHH students is described as an understudied area (Arfé, 2015), with recent work noting the need for continued research to inform effective writing instruction (Gärdenfors, 2023). These findings highlight an ongoing mismatch between the kind of responsive instruction that would best support deaf students’ writing development and the practices most commonly experienced and observed in classrooms.

### 1.2. Strategic and Interactive Writing Instruction

Positioning language diversity as an asset for literacy growth, Strategic and Interactive Writing Instruction (SIWI) provides a linguistically responsive framework specifically designed for deaf students, directed by their language needs and resources. SIWI is an approach to writing instruction for deaf students who use any language or modality to express themselves. The instruction is built upon cognitive and sociocultural frameworks with three foundational principles: (1) interactive instruction, (2) strategic instruction, and (3) metalinguistic knowledge/linguistic competence. Together, these principles provide a structure that supports writing development while deepening students’ linguistic awareness and ownership (K. Wolbers, 2008). Full descriptions and visual representations of the SIWI framework are available on the SIWI website, https://siwi.utk.edu/ (accessed on 14 November 2025), which hosts publicly accessible materials illustrating the model. These include a graphic summarizing the SIWI principles with brief ASL and English descriptions; an overview of instructional enactment with related visual scaffolds; an annotated model unit in video format; and the SIWI observation and fidelity instrument, which organizes instructional practices in a visual schema. Collectively, these materials illustrate the principal instructional processes and pedagogical features that define SIWI.

#### 1.2.1. Interactive Instruction

The SIWI principle of interactive instruction is grounded in sociocultural theories of learning (Lave & Wenger, 2003; L. S. Vygotsky, 1978; L. Vygotsky, 1994; Wertsch, 1991), which emphasize that knowledge develops through active involvement in social interaction during mediated activities. In the context of writing instruction, this approach involves multiple guided and shared writing experiences that allow students to write together, building on each other’s ideas and contributions. This dialogic pedagogy (Burbules, 1993; Wells, 2000) allows teachers to scaffold students’ learning in the moment, positioning the SIWI teacher as a responsive guide who actively facilitates the co-construction of text. The teacher uses these formative interactions to assess understanding, model strategies, and gradually release responsibility as students gain more independence (Englert et al., 2006; Pearson & Gallagher, 1983).

SIWI’s instructional process emphasizes the co-construction of text, in which teachers and students work together to compose writing with an authentic purpose and audience (Blanch et al., 2017; H. Dostal et al., 2015). In this way, students are apprenticed into the shared writing community, enabling them to identify as writers while gradually gaining independence in key writing skills (Williams, 2018; K. Wolbers et al., 2018). Unlike traditional writing instruction, which often assumes students possess sufficient foundational English skills, SIWI explicitly mentors deaf students in language use, providing real-time modeling and feedback.

#### 1.2.2. Strategy Instruction

The second foundational SIWI principle, strategy instruction, is informed by cognitive theories of writing and composing (Applebee, 2000; Flower & Hayes, 1980; Hayes, 1996, 2006), which view writing as a complex, goal-directed, and recursive process. These theories explain that learning to write effectively requires explicit instruction in the same cognitive strategies and self-regulatory processes that expert writers use while composing. Through explicit instruction, guided modeling, and an abundance of practice, students learn to internalize these strategies and apply them independently. During SIWI, teachers make this kind of thinking and these processes visually accessible and gradually transfer control of the writing process from teacher to student over time.

To support this process, SIWI teachers incorporate procedural facilitators such as cue cards or mnemonics (Scardamalia & Bereiter, 1986). These visual tools scaffold the learning process and help students to internalize strategies, gradually empowering them to self-regulate their processes and performance (Graham & Perin, 2007). For deaf learners, visual scaffolds have shown promise in supporting the acquisition of new skills (Skyer, 2023). Appanah and Hoffman (2014) found that when visual scaffolds such as rubrics were paired with guided interaction, deaf students were better able to internalize concepts and apply them independently. Those students who engaged in discussions with an adult while using the scaffold demonstrated the most significant growth in their writing. This suggests that these visual tools are most effective when paired with interactive, guided feedback.

#### 1.2.3. Metalinguistic Knowledge/Linguistic Competence

The third overarching SIWI principle consists of metalinguistic knowledge and linguistic competence. These are interrelated constructs grounded in theories of language acquisition, bilingualism, and translanguaging. Linguistic competence refers to an individual’s implicit, internalized knowledge of the structures of language, syntax, semantics, and phonology that enables natural language use (Chomsky, 1965, 2014; Jackendoff, 1994; Pinker, 1995). This competence develops not through direct instruction but through rich, interactive experiences in which individuals use language expressively and receptively to co-construct meaning with others. Through such communicative encounters, the mind unconsciously organizes complex patterns of linguistic form and meaning. In contrast, metalinguistic knowledge involves the conscious ability to reflect on and manipulate language as an object of thought (Gombert, 1992; Bialystok, 2001). These two dimensions of language knowledge are dynamically interconnected: linguistic competence provides the foundation for fluent expression, while metalinguistic awareness supports deliberate reflection and refinement of language use.

Translanguaging theory further broadens this view by asserting that multilingual and multimodal individuals draw from a single, integrated linguistic repertoire rather than separate language systems (García & Wei, 2014; Otheguy et al., 2015). Building on this theoretical foundation, García et al. (2017) describe how translanguaging can be enacted pedagogically to leverage students’ full linguistic repertoires for learning, providing a bridge between theory and practice that aligns with the instructional design of SIWI.

The SIWI framework translates these theoretical principles into classroom practice by creating conditions that nurture linguistic competence and metalinguistic awareness through implicit and explicit language learning (Ellis, 2015) and by applying translanguaging pedagogy in responsiveness to students’ diverse language experiences (K. Wolbers et al., 2023c). Opportunities for developing linguistic competence arise through authentic, interactive communication in which students express and receive ideas using ASL, written English, and other semiotic forms. These exchanges, such as collaborative text construction, peer dialogue, and meaning negotiation, allow students to engage in purposeful communication that naturally strengthens their implicit grasp of linguistic forms and functions. In contrast, metalinguistic awareness is fostered when teachers guide students to reflect on language explicitly: comparing ASL and English structures, including attention to how ASL’s visual–spatial grammar and syntax differ from English’s linear, inflectional system and how these contrasts can surface in writing, analyzing how meaning shifts across modalities, and considering how linguistic choices align with audience and purpose (Bialystok, 2001; Swanwick, 2017). For learners still developing expressive clarity, SIWI integrates multimodal strategies such as drawing, gesture, role play, and imagery to scaffold understanding and build conceptual connections (H. Dostal et al., 2019; Holcomb et al., 2023b). Their linguistic competence grows through meaningful language use, while their metalinguistic awareness deepens through deliberate reflection. Taken together, these approaches support the bilingual, biliterate, and cognitively flexible development of deaf students.

### 1.3. Motivation

Motivation is an essential component of writing development, influencing students’ engagement, persistence, and use of strategies while composing (Bruning & Horn, 2000; Pajares, 2003; Troia & Graham, 2002). Motivation warrants attention because writing instruction that is delivered without strong connections to authentic purpose, strategic support, or opportunities for student agency can diminish interest and stamina (Camacho et al., 2020; Magnifico, 2010; Troia et al., 2013). Motivated writers, by contrast, tend to seek feedback and show greater willingness to revise and refine their work (Martín et al., 2020; Waller & Papi, 2017).

Although motivation is not a standalone principle of SIWI, several mechanisms known to support writing motivation are embedded within its design. Sociocultural models emphasize that learners become more engaged when they participate in collaborative writing communities and co-construct text for meaningful audiences (De Smedt & Van Keer, 2018; L. S. Vygotsky, 1978). Cognitive models highlight the importance of explicit strategy instruction, guided practice, and opportunities to experience success with increasingly independent writing (Flower & Hayes, 1980; Graham & Harris, 2000). SIWI integrates both perspectives by supporting students through dialogic, interactive writing and by making visible the cognitive strategies used by proficient writers.

Emerging empirical evidence suggests that SIWI may contribute to enhanced writing motivation for deaf students. In a qualitative analysis of a year-long implementation, H. Dostal et al. (2015) documented increased willingness to write, stronger author identities, and greater initiative among middle school students participating in SIWI, alongside decreases in avoidance behaviors. These shifts were attributed to core SIWI features such as valuing students’ linguistic resources, embedding choice in writing tasks, requiring stated purpose and authentic audiences, and providing sustained guided instruction. These features aligned with theories emphasizing purpose and supportive scaffolding (Deci & Ryan, 1985; Guthrie & Humenick, 2004).

Quantitative intervention research provides additional, developing evidence. In the first SIWI randomized controlled trial, genre-specific writing motivation did not show statistically significant treatment differences; however, effect sizes indicated small to moderate positive impacts (0.33–0.54) favoring SIWI (K. Wolbers et al., 2022). This pattern aligns with qualitative findings suggesting that SIWI’s collaborative, purposeful, and strategically supported writing environment may bolster engagement and self-efficacy.

Related research examining elementary deaf students further demonstrates the importance of instructional context for writing motivation. K. Wolbers et al. (2019) found that English language competence, not hearing status, demographics, or ASL receptive skill, was the strongest predictor of writing motivation, highlighting the relevance of approaches like SIWI that integrate strategic support with opportunities to strengthen linguistic competence.

Theoretical grounding and emerging qualitative and quantitative evidence suggest a plausible rationale for examining motivation within the current replication study. Because motivation influences students’ willingness to participate in writing and persist through challenge, investigating SIWI’s effects on motivation is aligned with prior research on deaf students’ writing development.

### 1.4. Writing and Language Outcomes of SIWI

Across nearly two decades of research, SIWI has consistently demonstrated significant and sustained benefits for deaf students’ writing development in elementary and middle grades. Early studies employing quasi-experimental and single-case designs established SIWI’s promise by showing notable improvements in writing quality, as well as enhanced written language clarity and complexity. For instance, after eight weeks of instruction with middle school students (*n* = 33) at high levels of instructional fidelity, K. Wolbers (2008, 2010) reported significant gains in the treatment group for explicitly taught traits of information reports and untaught traits of narrative writing. These gains extended to contextual language measures from the Test of Written Language-3 and increased text length. Importantly, regardless of literacy backgrounds—ranging from emerging to proficient bilinguals—all students demonstrated significant growth across all measured writing and language variables (Cohen’s *d* = 1.27–2.65).

Further evidence supporting SIWI’s efficacy emerged from a study involving students in grades 3–5 (*n* = 63), who received instruction in personal narrative and persuasive writing over one semester (K. Wolbers et al., 2018). Despite teachers being novice SIWI implementers, with an average fidelity rate of 75% (lower than the previous study), students still exhibited statistically significant improvements in five out of six measured writing traits (Hedge’s *g* = 1.05–2.01). Additional gains were observed in words per t-unit for personal narrative writing (*g* = 1.13) and a word efficiency ratio measuring grammar clarity in phrasal and word strings across both genres (*g* = 1.06–1.09). Study findings underscore SIWI’s direct benefits for genre-specific traits and language development, as well as its broader impact on untaught genres (H. Dostal et al., 2021; H. Dostal & Wolbers, 2016).

Two single-case design studies further reinforced this positive trajectory. In one study, K. Wolbers et al. (2015) conducted five multiple-probe case studies across genre traits, identifying immediate and sustained improvements in information reports and persuasive writing among students with varying hearing levels (mild to profound) and diverse language profiles. In another study, K. Wolbers et al. (2020) utilized a multiple-baseline probe design to examine the impact of SIWI on elementary students’ written grammar and conventions (*n* = 6). This research revealed gains in word- and sentence-level language skills, including sentence length, verb tense usage, and punctuation accuracy.

A growing body of research highlights the importance of SIWI’s metalinguistic/linguistic principle in providing specialized writing instruction tailored to deaf learners’ needs for language development and knowledge. For example, K. A. Wolbers et al. (2014) found that SIWI significantly increased middle school students’ metalinguistic knowledge while reducing the presence of ASL grammatical features naturally appearing in their writing (K. A. Wolbers et al., 2014). Similarly, exposure to SIWI led to substantial decreases in unintelligible utterances (H. Dostal & Wolbers, 2014) and phrasal errors (Bowers et al., 2018) in written English, alongside greater linguistic competence. In H. Dostal and Wolbers (2014), it was observed that 23 deaf students in grades 4–6 demonstrated growth in expressive language complexity through increased mean length of ASL utterances after participating in SIWI. Furthermore, a quasi-experimental study involving 69 deaf students in grades 3–6 revealed significant gains in students’ signed or spoken compositions, which incorporated recount and information report traits, alongside simultaneous improvements in their written language. Notably, these advancements in expressive language occurred without explicit instruction targeting expressive language composing, underscoring the broader impact of SIWI on students’ overall language development (K. Wolbers et al., 2024).

Students in these studies were primarily ASL–English bilinguals, although monolingual students and some using additional home languages (spoken or signed) also participated. Due to varied early language access, students’ proficiency in ASL and English ranged significantly from severely delayed to age-appropriate language. They were educated across a range of school contexts, including ASL–English bilingual programs and settings using sign-supported speech; in some studies, listening and spoken language programs were also represented. These studies demonstrate SIWI as beneficially impactful to deaf students’ writing quality and clarity, language development, and motivation regardless of language background and setting.

Interestingly, parallel benefits have been documented for teachers involved in SIWI research and professional development initiatives. Educators demonstrated significantly enhanced knowledge of evidence-based writing practices, increased pedagogical efficacy, and greater enthusiasm for teaching writing to deaf students (Graham et al., 2021; K. Wolbers et al., 2023b).

### 1.5. Broader Impacts

A number of studies conducted outside the development team demonstrate the global relevance of SIWI as an effective writing program for deaf students. Favorable outcomes have been observed across various age groups and regions: narrative writing improvements in US elementary students (Aboudi, 2022), sentence writing advancements in Thailand (Pimwong & Saksiriphol, 2018), middle grades progress in Indonesia (Huda et al., 2023), secondary and postsecondary achievements in Saudi Arabia (Al-Enazi & Turkestani, 2024; Alsraisry & Albakheet, 2020), and enhanced writing skills among deaf adults in Iran (Kolaqani et al., 2022).

Beyond writing and language outcomes, SIWI has contributed to literacy development in areas such as spelling (Simpson, 2022; Villanueva, 2022) and reading proficiency (Skerrit, 2017). These findings highlight its versatility as a tool for improving overall literacy skills. Implementation of SIWI has also expanded into new contexts. Speech-language therapists have successfully used it for individualized or small group instruction (Secora et al., 2023). Early elementary students have benefited from its adaptation into a sign-composing framework called Strategic and Interactive Signing Instruction (SISI; Holcomb, 2024). Lastly, a language evaluation tool used for monitoring and setting language goals for SIWI has been developed and applied to the writing of deaf Chinese students (Cui, 2022). These global applications and extensions emphasize the broad impact of SIWI on deaf education and reinforce the need for a large-scale study to validate its effectiveness across diverse contexts.

### 1.6. RCT and Replication

Building upon a robust foundation of evidence that SIWI yields positive effects, the first randomized controlled trial was conducted with 15 teachers and 79 students (K. Wolbers et al., 2022). Results revealed a statistically significant advantage for students receiving SIWI compared to those undergoing business-as-usual (BAU) instruction. The most substantial gains were observed in recount (*g* = 3.32) and information report writing traits (*g* = 1.12), which were maintained at follow-up assessments (*g* = 3.12 and *g* = 0.62, respectively). Treatment effects were also notable for written language clarity and complexity (*g* = 0.44–0.71). Over one academic year, students exposed to SIWI achieved an average progress equivalent to 1.2 grade levels in broad written language skills, as measured by the Woodcock–Johnson IV, while BAU group students showed negligible changes (*g* = 1.88). The current replication study is aimed at examining the effects of SIWI with a larger and more diverse sample of deaf students across the United States. Replication plays a crucial role in establishing a reliable evidence base in education. By validating or refuting initial findings, replication reduces bias and bolsters confidence in the observed effects (Cook, 2014). This process is particularly vital in specialized fields like deaf education, where research often involves smaller sample sizes and diverse contexts and populations. Travers et al. (2016) argue that replication should not be dismissed as redundant; rather, it serves as a systematic approach to determining whether intervention outcomes are reproducible and generalizable across varied participants, settings, and conditions. For interventions such as SIWI, which cater to heterogeneous populations and learning environments, replication ensures that the benefits observed in one study can be extended to broader contexts.

### 1.7. The Current Study

Funded by the Institute of Education Sciences (IES), this project represents the second RCT of SIWI and the first nationwide replication conducted in upper elementary grades. The primary objective was to evaluate whether the significant improvements in writing quality and language clarity observed in prior research could be replicated across diverse instructional contexts and varying language use among students. To achieve this, we utilized genre-specific, trait-based rubrics alongside the Structured Analysis of Written Language (SAWL) to assess students’ recount and information report writing. Additionally, standardized measures of written language proficiency were employed using the Woodcock–Johnson IV broad written language composite, complemented by genre-specific motivation surveys administered at both the beginning and end of the academic year. By replicating previous findings across varied educational settings, this study aims to reinforce the evidence base for SIWI and provide deeper insight into how structured, language-rich writing instruction can effectively support literacy development of deaf students.

## 2. Method

### 2.1. Research Questions

Our main research question is as follows: To what extent does participating in SIWI improve student writing, language, and motivation compared to a business-as-usual (BAU) learning condition? Our hypothesis is that students who receive SIWI for recount and information report writing will demonstrate more growth than students in BAU condition with genre-related writing traits, written language clarity, and genre-specific writing motivation. An exploratory question of this study asks: Does the treatment effect differ depending on students’ handwriting fluency?

### 2.2. Design

This nationwide replication study was conducted over two academic years (2018–2020) to obtain enough participants to meet our power analysis^1^ (*n* = 50 teachers; > 200 students). Deaf students make up only 1.4% of the student body within K-12 classes in the US (Khalsa & Chan, 2023). Thus, we included schools and teachers from all regions of the continental US in this study. There were a total of 294 students who were randomly assigned at the teacher level to BAU or treatment groups. There were a total of 50 teachers and 20 different schools, providing an average of 5–6 students per teacher and 14–15 students per school. Writing instruction for both groups was implemented for one academic year; measures were collected at the beginning and end of the school year.

#### 2.2.1. Random Assignment

Teachers and their students were randomly assigned to BAU and treatment groups through a computer-generated randomization process. At locations where there was more than one participating teacher, an equal number of teachers were assigned to BAU and SIWI conditions. We implemented a waitlist control design; therefore, initial group sizes were intentionally not equal. To ensure equitable access to SIWI and to reduce the risk of student crossover between BAU and SIWI teachers within a location, we randomized a larger number of teachers to BAU in Year 1, with the plan that these teachers would move into the treatment condition in Year 2. As a result, Year 1 included 91 students (17 teachers) in BAU and 74 students (13 teachers) in the treatment group; in Year 2, 37 students (7 teachers) were in BAU, and 92 students (13 teachers) were in the treatment group. The sample reflects a planned asymmetry rather than an imbalance in the randomization process itself.

#### 2.2.2. Study Disruption Due to COVID-19

In the second year (when treatment group numbers were highest), the study was impacted by school closures in late February and early March due to COVID-19. Instruction was shortened by 3–4 months, and post-data collection administration was changed (described under data collection). During one academic year of writing instruction, teachers typically covered three different genres of writing—recount, information report, and persuasive—as aligned with later elementary writing standards as published in the Common Core State Standards (National Governors Association Center for Best Practices & Council of Chief State School Officers, 2010). In the year of COVID-19, the majority of teachers did not teach persuasive writing, or they taught it unconventionally online and/or sporadically, so these data were not included. Recount instruction was completed, and information report writing instruction was differentially completed across classrooms. Those schools that started the academic year prior to Labor Day had largely finished information report writing instruction, while those that started after Labor Day had a few to several weeks of instruction remaining. We include the data sets for both of these genres in the study due to students receiving the full intervention for recount and most of the intervention for information report writing.

#### 2.2.3. Funding and Participant Compensation

Funding was provided for this project from the IES through grant R324A170086. Compensation was offered to participants for one year of involvement in the amount of $1000 to BAU teachers and $1500 to SIWI teachers. Teachers in both groups additionally received $100 for every complete student data set that was collected. If school policy allowed it, a stipend equivalent to $100 per student was paid directly to the school for their role in supporting administrative tasks such as coordinating data assessors and distributing information to caregivers.

Both BAU and SIWI teachers committed to at least 2–2.5 h a week in writing instruction during their participation. Teachers in the BAU group video-recorded a unit of their typical writing instruction, which was submitted to the research team. SIWI participants attended a week-long professional development program in Tennessee prior to the start of the school year and attended online coaching with a SIWI team member eight times during the academic year. They videotaped all writing instruction using a tablet provided to them, which allowed for automatic upload via the SWIVL platform to a secure area that could be accessed by the researchers. Teachers from both groups completed a survey about their writing instruction practices.

### 2.3. Teacher Participants

The treatment group included 26 teachers, and the BAU group included 24 teachers. Teachers in both groups provided self-reported demographic and professional information collected at the beginning of the year through an online survey. Table 1 presents demographic information for teachers in the SIWI and BAU treatment conditions.

Treatment Group. All teachers in the treatment group identified as female. Two teachers identified as people of color (one Black and one Asian/Pacific Islander), while the remaining teachers identified as White. Four teachers identified as Deaf, two of whom reported using hearing aids; the others identified as hearing. Four teachers were native users of ASL, and the remainder had learned ASL later in life. Most teachers held a master’s degree, with others reporting bachelor’s (*n* = 5) or advanced graduate degrees (*n* = 2). On average, teachers had over a decade of experience in the field. Nineteen teachers worked in bilingual/multilingual programs using ASL and English, while seven worked in programs emphasizing spoken English with or without signs. Most teachers described their preparation to teach writing prior to SIWI as adequate (*n* = 18), with a few indicating exceptional (*n* = 3) or minimal (*n* = 5) preparation. At the start of the study, 20 teachers reported using a writing curriculum, most frequently Writer’s Workshop, Framing Your Thoughts, McGraw Hill Wonders, and Houghton Mifflin Harcourt Journeys. One teacher withdrew midyear due to a change in position.

BAU Group. The BAU group consisted of 23 female teachers and one male teacher. One teacher identified as Black, and the remainder identified as White. Four teachers identified as Deaf and reported using hearing aids. As in the treatment group, four were native ASL users, and the others learned ASL later. Most teachers held master’s degrees, with others reporting bachelor’s (*n* = 3) or advanced graduate degrees (*n* = 4). Their average years of teaching experience were comparable to the treatment group. Nineteen teachers worked in bilingual or multilingual programs using ASL and English, while six worked in programs emphasizing spoken English. Most described their preparation to teach writing as adequate (*n* = 18), with a few rating it as exceptional (*n* = 4) or minimal (*n* = 2). Eighteen teachers reported using a writing curriculum at the start of the study, naming the same programs most often cited by the treatment group. One teacher withdrew midyear due to being overcommitted.

Teachers across both groups represented a range of educational settings, with just over half employed at schools for the deaf and the remainder working in local education agencies serving deaf and hard-of-hearing students in self-contained or pull-out classrooms.

### 2.4. SIWI Professional Development

The SIWI professional development (PD) program is designed to strengthen teachers’ pedagogical and content knowledge through an intensive, sustained model of learning (Darling-Hammond & Richardson, 2009). Its purpose is to help teachers internalize and apply the core SIWI principles—strategic instruction, interactive instruction, and the development of metalinguistic knowledge and linguistic competence—through a blend of simulated experiences, authentic classroom applications, and continuous, context-based feedback.

Teachers assigned to the treatment condition began their participation with a week-long summer institute. This workshop followed iterative cycles of learning, practice, and feedback, with each cycle building upon the last to provide comprehensive exposure to the SIWI framework. By the end of the week, teachers had developed plans for implementing SIWI in their classrooms and introducing its routines to their students.

After roughly two months of classroom application, participants reconvened for a two-day follow-up workshop focused on analyzing student writing and planning instruction for a new writing genre. Throughout the academic year, teachers also engaged in eight individualized virtual coaching sessions via Zoom to reinforce and refine their implementation. In addition, SIWI coaches conducted two on-site visits to each classroom, except during spring 2020, when the onset of COVID-19 disrupted in-person school operations.

### 2.5. SIWI Intervention

Teachers implementing SIWI provided instruction in recount and information report writing genres for approximately 18 h over nine weeks per genre, except during the pandemic disruption, which reduced the overall hours of instruction for information report writing. Instruction was grounded in SIWI’s core principles—strategic and interactive instruction and the development of linguistic/metalinguistic knowledge—and these principles guided planning, teaching, and reflection across units. Writing lessons involved co-constructing text within guided, interactive contexts that included both shared and independent writing opportunities. Teachers emphasized authentic purposes and audiences for writing, modeled and scaffolded strategies for engaging in the writing process (including genre-specific features and skills), and used translanguaging pedagogies to clarify and extend students’ linguistic knowledge. Additional details on SIWI principles and the SIWI Observation and Fidelity Instrument are available at https://siwi.utk.edu/ (accessed on 14 November 2025).

#### Fidelity Scoring

One unit per teacher per genre was selected and scored for instructional fidelity. Each unit represented a full cycle of writing instruction—from identifying a purpose and audience to publishing and sharing—and typically spanned five to eight lessons. Instructional fidelity was assessed using the SIWI Observation and Fidelity Instrument (H. Dostal & Wolbers, 2015), which includes 53 indicators organized under three SIWI principles: strategic (e.g., explicit discussion of text structure), interactive (e.g., peer collaboration), and metalinguistic/linguistic (e.g., strategies for achieving shared understanding of language). Items were rated as fully (1), partially (0.5), or not implemented (0), and total scores were converted to percentages to represent overall fidelity.

Twenty percent of the units were double-coded by four research team members to establish interrater reliability (ICC = 0.87). Coders then met to reach consensus on final scores; the remaining units were coded by one team member.

Treatment teachers’ fidelity scores ranged from 47% to 90%, averaging 71% for recount writing and 70% for information report writing. These levels are consistent with prior SIWI professional development research, which shows that first-year SIWI teachers average around 75% fidelity and typically exceed 90% with continued implementation for three years (K. Wolbers et al., 2016). Previous studies have shown that teachers positively impact students’ writing and language outcomes even as they work toward higher implementation fidelity (K. Wolbers et al., 2018, 2022).

### 2.6. BAU Writing Instruction as Distinct from SIWI

To document and compare instructional practices, researchers administered a 26-item survey at the beginning and end of the school year. Items, presented in random order, represented (a) evidence-based writing instruction (7 items), (b) evidence-based writing supports (8 items; Brindle et al., 2016), (c) deaf education practices consistent with SIWI (7 items; Strassman & Schirmer, 2013; Williams & Mayer, 2015), and (d) non-SIWI-aligned deaf education practices (4 reverse-scored items). Teachers rated frequency of implementation on an 8-point Likert scale (1 = never to 8 = several times a day). These ratings were confirmed through the videotaped instruction submitted to the research team.

Independent samples t-tests were conducted to compare groups. At pretest, no significant difference was found between treatment (*M* = 4.16, *SD* = 0.80) and BAU (*M* = 4.44, *SD* = 0.54) teachers, *t*(48) = −1.45, *p* = 0.15. At posttest, treatment teachers reported significantly greater use of evidence-based practices (*M* = 5.06, *SD* = 0.68) than BAU teachers (*M* = 4.19, *SD* = 0.68), *t*(46) = 4.43, *p* < 0.001. Treatment teachers also showed reduced use of non-SIWI-aligned practices, patterns not observed in the BAU group.

### 2.7. Student Participants

A total of 294 deaf students in grades 3 through 6 participated in the study. Of these, 74 (44.6%) participated in 2018–2019 and 92 (55.4%) in 2019–2020 within the treatment group (total *n* = 166), while 91 (71.1%) participated in 2018–2019 and 37 (28.9%) in 2019–2020 within the BAU group (total *n* = 128). Participants were recruited from schools for the deaf, public schools, and mainstream programs across the United States and were randomly assigned at the class level to either the SIWI intervention group (*n* = 166) or a BAU control group (*n* = 128). Participants represented broad distribution across elementary grade levels: 17% of students were in grade 3 (*n* = 49), 24% in grade 4 (*n* = 72), 27% in grade 5 (*n* = 81), and 31% in grade 6 (*n* = 91). One participant did not report grade level. Mean ages correspond closely with expected ages for each grade level. The mean age for students in grade 3 was 8 years and 9 months, for grade 4 it was 9 years and 9 months, for grade 5 it was 10 years and 11 months, and for grade 6 it was 11 years and 10 months. One student, whose grade was unreported, was 11 years and 9 months old.

The sample was diverse by gender, race, and ethnicity. Forty-three percent of participants reported identifying as female (*n* = 126), 56% as male, and 1% did not report gender (*n* = 2). In terms of racial and ethnic background, 41% of students identified as White (*n* = 120), 23% as African American (*n* = 69), 20% as Latine (*n* = 59), 6% as Asian or Pacific Islander (*n* = 19), 1% as Native American (*n* = 1), 5% as multiracial (*n* = 15), and 4% as another racial or ethnic identity (*n* = 11). Across both groups, approximately 22% of students were identified as having an additional disability (*n* = 66). The most commonly reported disabilities included ADHD (*n* = 16), cognitive impairment (*n* = 6), and cerebral palsy (*n* = 5). Table 2 presents demographic information for students in the SIWI and BAU treatment conditions.

### 2.8. Educational Context

Students in the study were from a range of educational settings serving deaf learners across the United States. Among those assigned to the treatment group, 69.3% (*n* = 115) attended schools for the deaf, 28.3% (*n* = 47) were enrolled in self-contained classrooms in public schools, and 2.4% (*n* = 4) received itinerant services. The distribution for the BAU group was similar, with the largest number of students, 57% (*n* = 73), enrolled in schools for the deaf, then those in self-contained public school classrooms, 34.4% (*n* = 44), and the remaining 8.6% (*n* = 11) receiving itinerant support.

Language and communication philosophies varied across programs. Within the treatment group, the largest proportion of students, 41.6% (*n* = 69), were educated in ASL/English bilingual settings, followed by 30.7% (*n* = 51) who attended total communication programs where ASL was integrated. An additional 15.1% (*n* = 25) were in total communication programs using simultaneous communication, and 12.7% (*n* = 21) were educated in listening and spoken language environments. In the BAU group, 30.5% (*n* = 39) of students were enrolled in ASL/English bilingual settings, 33.6% (*n* = 43) in total communication programs incorporating ASL, 29.7% (*n* = 38) in programs using simultaneous communication, and 6.3% (*n* = 8) in listening and spoken language programs.

Students’ language proficiency in both American Sign Language (ASL) and spoken English was evaluated using a five-point Likert scale. On this scale, a score of 1 represented the ability to “express most anything,” while a score of 5 indicated that the student “does not express anything in the language.” Ratings were provided by teachers who were fluent in the language(s) they evaluated. Based on these ratings, an overall language proficiency variable was developed to represent each student’s highest proficiency in either ASL or spoken English. This combined language proficiency measure, rather than separate assessments by modality, has been identified as a strong predictor of literacy outcomes (K. Wolbers et al., 2025). See data in Table 3.

Attendance patterns were comparable across groups, though rates of absenteeism were somewhat high, likely reflecting the impact of COVID-19 disruptions and school closures during the study. In the treatment group, 79.5% (*n* = 132) of students missed fewer than ten school days, while 13.9% (*n* = 23) missed between ten and twenty days, and 6.6% (*n* = 11) were absent for more than twenty days. A comparable pattern emerged in the BAU group, where 81.3% (*n* = 104) missed fewer than ten days, 10.9% (*n* = 14) missed between ten and twenty days, and 7.8% (*n* = 10) missed more than twenty days.

### 2.9. Hearing Levels

Hearing levels, measured in the better ear without amplification, ranged from typical to profound across participants. These levels indicate the students’ access to spoken language without devices; students with slight to moderate hearing levels may access a few to many speech sounds, whereas those with moderately severe to profound hearing levels are unlikely to hear speech around them. In the treatment group (*n* = 166), 7 students (4.2%) had mild hearing levels (26–40 dB), 22 (13.3%) had moderate hearing levels (41–55 dB), 27 (16.3%) had moderately severe hearing levels (56–70 dB), 30 (18.1%) had severe hearing levels (71–90 dB), and 73 (44%) had profound hearing levels (91+ dB). Information was unavailable for 7 students (1.8%). In the BAU group (*n* = 128), 3 students (2.3%) had typical to slight hearing levels (0–25 dB), 3 (2.3%) had mild hearing levels (26–40 dB), 10 (7.8%) had moderate hearing levels (41–55 dB), 27 (21.1%) had moderately severe hearing levels (56–70 dB), 27 (21.2%) had severe hearing levels (71–90 dB), and 57 (44.5%) had profound hearing levels (91 dB or greater); hearing level information was unavailable for 1 student (0.8%).

In the treatment group, 48 students (28.9%) did not have amplification devices, while others used a range of devices. Sixty students (36.1%) used hearing aids, 11 (6.6%) had one cochlear implant, 17 (10.2%) used a cochlear implant in one ear and a hearing aid in the other, and 29 (17.5%) used bilateral cochlear implants. Amplification device data were missing for 1 student In the BAU group, 24 students (18.8%) did not have amplification. Among those who did, 58 (45.3%) used hearing aids, seven (5.5%) had one cochlear implant, 15 (11.7%) used a cochlear implant and a hearing aid, and 23 (18.0%) used bilateral cochlear implants. Amplification information was unavailable for one student.

We report students’ best hearing levels based on either their amplified hearing (for those who used their hearing devices frequently or always) or their unamplified hearing levels (for those who did not have hearing devices or those who wore them infrequently to never). In the treatment group, 20 students (12%) were identified as having slight hearing levels (dB reported above), 20 (12%) had mild hearing levels, and 11 (6.6%) had moderate hearing levels. Twelve students (7.2%) were reported to have moderately severe hearing levels, 11 (6.6%) as severe hearing levels, and 45 (27.1%) as profound hearing levels. Information was not available for 42 (25.3%) of students. In the BAU group, 17 students (13.3%) had typical to slight hearing levels, 16 (12.5%) had mild hearing levels, 13 (10.2%) had moderate hearing levels, 8 (6.3%) had moderately severe hearing levels, 10 (7.8%) had severe hearing levels, and 32 (25%) had profound hearing levels. Information was not available for 31 (24.2%) of students.

No demographic variables demonstrated meaningful imbalance across conditions.

### 2.10. Data Collection

Data were collected from student participants at the beginning and end of the academic year. All assessments at the beginning of the year were collected before teachers began writing instruction. Local data collectors, trained by a member of the research team, collected the majority of the pre- and post-assessments, with the exception of writing samples, which were collected by the classroom teachers. Once the research team received the writing samples, they were typed, coded, and blinded for scoring. An external evaluator was hired to confirm the data collection and processing procedures and ensure objectivity measures were in place.

In the spring of 2020, school closures due to the COVID-19 pandemic significantly disrupted end-of-year data collection, which was conducted primarily online by teachers. Despite efforts to reach all participating students, post-assessments could not be completed for a substantial portion of the sample, resulting in a 30% decrease in data collection compared to pre-assessments. This disruption disproportionately affected students in the SIWI treatment group due to the waitlist control design of the study. Specifically, this design led to a higher number of BAU participants during the first year and a larger cohort of treatment participants during the second year, coinciding with the onset of the pandemic. Consequently, approximately 55% of treatment group participants were impacted by the reduced data collection, compared to 29% of those in the BAU group.

### 2.11. Measures

#### 2.11.1. Genre-Related Writing Traits

To assess the impact of SIWI on writing proficiency, students were given prompts similar to the 4th-grade National Assessment of Educational Progress (NAEP; National Assessment Governing Board, US Department of Education, 2010) for recount and information report writing. Prompts A and B for each genre were randomly assigned to half of the treatment and BAU groups and then rotated for post-data collection. Students were given 20 min to respond to each prompt. The prompts asked students to convey a personal experience through writing (e.g., sharing about an event from their childhood or a memorable experience at school) or to inform the reader on a topic (e.g., explaining a hobby, place, or animal).

Research team members and trained graduate assistants scored the writing samples using genre-specific trait rubrics developed during a prior IES development grant. These rubrics were informed by the NAEP 2011 holistic scoring guidelines. Each rubric used a six-point scale and evaluated three primary traits: opening, content, and organization. The opening and content traits were genre-specific. For recount writing, the opening trait assessed students’ orientation to the event, and the content trait evaluated the clarity and sufficiency of events. For information report writing, the opening trait assessed the clarity of the topic, and the content trait evaluated the quality and relevance of facts. The organization trait was consistent across genres and captured the connection between ideas, overall text structure, and use of transitions. Interrater reliability for at least 20% of the samples in the first year was 0.936 ICC for recount and 0.964 ICC for information report, and 0.985 and 0.964, respectively, in the second year.

#### 2.11.2. Written Language Clarity

Students’ recounts and information report writing samples were evaluated for grammatical clarity and efficiency in written English using the Structural Analysis of Written Language (SAWL; White, 2007). The SAWL is designed to provide a quantitative measure of deaf students’ syntax and allows for comparisons of writing samples across time. When scoring with the SAWL, the writing is divided into T-units—independent clauses and their dependent clauses. Each T-unit is rated as perfect (no errors) or flawed (minor errors but complete structure). If a T-unit is neither, the scorer evaluates smaller error-free word strings of three or more words; any leftover words are ignored. The Word Efficiency Ratio (WER III) is then calculated by dividing the total number of words in perfect T-units, flawed T-units, and perfect word strings by the total number of words in the sample. Members of the research team and graduate students scored the samples. Interrater reliability for at least 20% of the samples was 0.984 ICC.

#### 2.11.3. Broad Written Language

Students’ writing performance was also assessed using the broad written language cluster from the Woodcock–Johnson IV Tests of Achievement (WJ-IV; Schrank et al., 2014). The broad written language cluster includes three subtests (i.e., Spelling, Sentence Writing, and Writing Samples), which collectively capture students’ orthographic knowledge, syntactic development, and ability to generate written responses. Each subtest provides a standard score and a W-score. The standard score is norm-referenced (*M* = 100, *SD* = 15) and allows for comparison to national norms, with any score below 40 reported as 0. The W-score, derived from Rasch modeling, represents performance on an equal-interval scale and is not truncated, providing a sensitive measure of growth over time. The broad written language cluster was administered at the beginning and end of the school year by trained data collectors or teachers (when necessary due to COVID-19 limiting access), following standardized administration protocols. Research team members and graduate assistants scored the subtests and entered results into Riverside Score, the digital scoring platform for the WJ-IV, which automatically generated standard and W scores.

#### 2.11.4. Writing Motivation

Students’ writing motivation was assessed using the Writing Activity and Motivation Scale (WAMS, Troia et al., 2013). The WAMS includes sections specific to the recount and information report genres, each including 15 items rated on a 0–7 scale from totally disagree to totally agree, with an aggregate internal consistency reliability of α = 0.88 as obtained using Cronbach’s coefficient alpha. Each section begins with a scenario so that students can contextualize the questions within each genre. Items measure theorized constructs of motivation, including one’s self-efficacy (e.g., “I would be able to find mistakes and confusing or weak spots in my story and change them to improve my work.”) and the task interest or value (“Writing articles like this is important for my education and life.”). The WAMS was administered at the beginning and end of the school year to capture changes in students’ genre-specific writing motivation over time.

To ensure accessibility and consistency of administration, the survey was provided in ASL, spoken English, and written English, with ASL video-recorded and spoken English audio-recorded to ensure standardization across sites. Data collectors noted surveys from students who did not appear to understand the motivation survey or who responded carelessly (e.g., circling answers without reading or watching the question). Approximately 20% of the data was excluded for these reasons.

#### 2.11.5. Handwriting

Handwriting fluency has been found to be causally related to writing quality and fluency (Santangelo & Graham, 2016), and thus, a measure of handwriting fluency was included at the beginning and end of the year. Analyses including the handwriting variable are exploratory; there are no prior SIWI studies that included handwriting fluency. It is measured by the number of words students can copy without any mistakes from short paragraphs found in the copying subtest from the Group Diagnostic Reading Aptitude and Achievement Tests (Monroe & Sherman, 1966) in 90 s (Graham et al., 1997). Reliability for this measure is 0.99. 

### 2.12. Data Analysis

#### 2.12.1. Treatment Analyses

The analysis model is pre-post regression for students (s) nested within classrooms (c). The conceptual form of the model is given by the following equation:

*Post_sc_* = *Intercept_sc_* + *ClassPre_c_* + *StudentPre_sc_* + *SIWI_c_* + *StudentPre_sc_* * *SIWI_c_* + *e_sc_*

where *Post_sc_* is the outcome score, *ClassPre_c_* is the grand-mean centered pretest score for the classroom, *StudentPre_sc_* is the deviation pretest score for the student within the classroom, *SIWI_c_* is the randomly assigned classroom treatment indicator variable, and StudentPre_sc_ * SIWI_c_ is the interaction of treatment with student pretest. We set up this model to allow for the effect of pretest upon posttest to be different for classrooms versus students within classrooms (August et al., 2009). Regression slopes for SIWI will indicate the extent to which students in treatment classrooms outperform students in BAU classrooms. The interaction will indicate the extent to which treatment effects may differ based on student pretest scores (i.e., how pretest might moderate treatment efficacy). Models were fit in SAS PROC MIXED 9.4 (Littell et al., 2006) using the Kenward–Roger method for degrees of freedom for small samples (Faes et al., 2009; McNeish, 2017). We used default REML in PROC MIXED, which assumes missing at random for missing data—we later report two-level full information maximum likelihood (FIML) estimates from Mplus as a sensitivity check.

ICCs were computed for all outcomes to quantify the proportion of variance attributable to clustering at the classroom level (see Table A1 in Appendix A). ICCs for the primary outcomes ranged from 0.31 to 0.55, indicating substantial between-classroom variance. Exploratory outcomes showed ICCs from 0.04 to 0.28, reflecting small to moderate clustering. The handwriting outcome showed moderate ICCs (0.27–0.42). These values justify the use of multilevel modeling for a cluster-randomized literacy intervention.

Additionally, due to the small sample of teachers, we analyzed the students within classrooms as a two-level model. As a sensitivity check, we also analyzed the data as a three-level model of students within classrooms within teachers (to account for the repetition of 11 teachers participating in both years). The results for all estimates were essentially identical. We therefore report the simpler two-level model.

#### 2.12.2. Moderation Analyses

As exploratory analyses, three outcomes were chosen to evaluate how handwriting fluency might moderate the treatment effect. This model is the same as the treatment analysis, but with an additional main effect of handwriting fluency and an additional interaction of handwriting fluency with assigned treatment. Poorer handwriting may inhibit writing fluency and one’s ability to demonstrate their writing knowledge in handwritten samples, whereas stronger handwriting may allow a student to fluently express their knowledge of writing process and genre through written samples.

## 3. Results

Table 4 shows descriptive statistics for each outcome variable as well as handwriting fluency. Values are shown by wave (pre and post) and by treatment group. The writing total variable combines the scores for three writing traits (rated from 0 to 6) for a total maximum score of 18. Writing means for both groups were between 3 and 4 points at pre-assessment and diverged by post-assessment. The WER III is a ratio of correct grammar structure, ranging from 0 to 1. Scores across both groups were approximately 0.7, demonstrating considerable room for growth in language clarity. The W-score is reported for the WJ-IV broad written language. The W-score is a developmentally scaled score, and both group means at pretest were 461. Each genre-based motivation score is an average of its 15 survey items, ranging from 0—totally disagree to 6—totally agree. Mean motivation scores of 4 points indicate students “agree a little” with the motivation items. Handwriting is reported as the number of words correctly written in 90 s.

### 3.1. Results of Treatment Analyses

An important consideration in treatment analyses is baseline equivalence (What Works Clearinghouse, 2022). Table 5 shows the effect size (Cohen’s *d*) for the SIWI treatment versus BAU for each outcome at baseline. All outcomes except one (Info Motivation) were within acceptable limits for baseline equivalence (d ≤ 0.05) or statistical adjustment (0.05 < d < 0.25) using the pretest score (What Works Clearinghouse, 2022).

Table 6 shows results for the multilevel treatment analysis. For each outcome test, the estimates for the variables in the analysis equation (fixed effects) are provided, with an effect size for the treatment fixed effect, and random effects for the variability due to classrooms and measurement error (residual). The fixed effect is a model-based estimate of the difference due to treatment, standardized by the residual *SD* (Hedges’s *g*; Hedges, 2007). Hedges’s *g* is akin to Cohen’s *d* but is model-based, indicating the number of adjusted *SD*s the treatment group could be expected to outperform the BAU group.

Due to the extent of missing data (see Table 4), we replicated the analyses in Mplus as a bivariate two-level path model with FIML estimation and found the effect sizes to be essentially the same as the regular two-level regression model. We therefore report the simpler regression estimates. See Table A2 in Appendix A for FIML estimates.

The first outcome of recount writing had a model-predicted treatment effect of 2.37 units (*p* < 0.01). This means that, on average, students who received SIWI scored 2.37 points higher on their post-samples compared to BAU students, once prior writing ability (pretest) was accounted for. The standardized effect size was large at 1.11. The second outcome of info writing had a model-predicted treatment effect of 1.38 units (*p* < 0.01). Again, SIWI students outperformed BAU students on information report writing by 1.38 points at the end of the year, after accounting for pretest differences. The effect size was also large at 0.72. The third outcome, recount WER III, was statistically significant (*p* = 0.02) with a model-predicted treatment effect of 0.09, indicating SIWI students’ post-samples demonstrated greater grammatical clarity by 9% in their recount writing compared to BAU students after accounting for pretest differences. There was a moderately large effect size of 0.64. Although not statistically significant (*p* = 0.36), grammatical clarity on the info WER III outcome showed a 0.02, or a 2%, advantage for the treatment group in information report writing with a small effect size of 0.20. Similarly, the fifth outcome, WJ-IV broad written language, was not statistically significant (*p* = 0.15) but did show a small effect size of 0.27. The final two outcome variables, recount motivation (*p* = 0.03) and info motivation (*p* = 0.02), were statistically significant with model-predicted treatment effects of 0.31 and 0.36, respectively. This means that, on average, students who received SIWI were a third of a point higher (on a 6-point scale) than BAU students at posttest after accounting for pretest motivation. The standardized effect sizes were moderate at 0.49 and 0.47.

Overall, the effect sizes were positive, ranging from 0.20 to 1.11, suggesting promise for the efficacy of the SIWI program across writing, language, and motivation outcomes.

### 3.2. Results of Moderation Analyses

Table 7 shows the moderation analyses for three outcomes: recount writing, info writing, and broad written language. Table 7 is laid out in the same way as Table 6, but with two additional fixed effects: the main effect of handwriting fluency and the interaction of handwriting fluency with treatment. These two rows can be evaluated for the size of the estimates as well as their statistical significance.

Because interaction effects can be difficult to interpret, we used these estimates in SAS release 9.4 PROC PLM (SAS Institute Inc., 2014, Cary, NC USA) to produce graphs of the model-predicted relation between handwriting fluency and the outcome, separately by treatment group. In these graphs, the vertical axis shows the model-predicted outcome score, and the horizontal axis shows the grand-mean centered value for handwriting fluency (see Table 4). The lines are labeled for the model-predicted relation for SIWI and BAU, with 95% confidence limits shaded around the lines.

**Figure 1:**
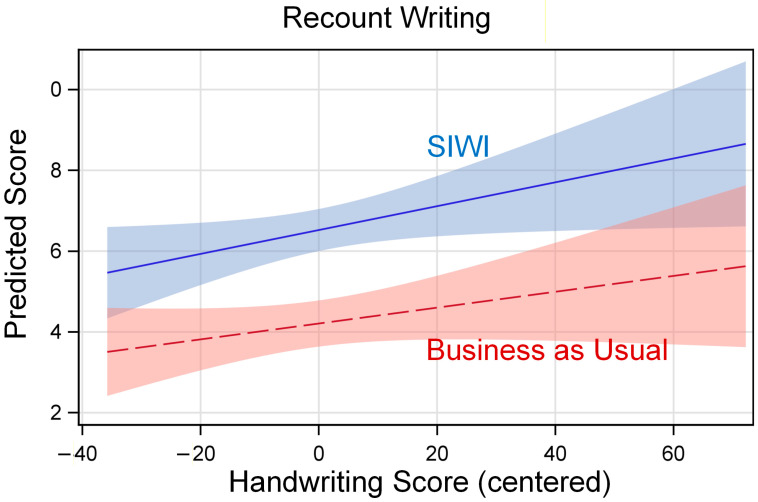
Moderation for Recount Writing by Handwriting Fluency. Note. Prediction lines are based on the multilevel moderation analysis (Table 7), with 95% confidence limits produced with SAS PROC PLM.

Figure 1 shows the relation between handwriting fluency and recount writing. In Figure 1, the lines slope upward, suggesting that there is a positive relation between handwriting and recount writing. The SIWI line is above but parallel to the control line, suggesting that the positive treatment effect is strong and uniform (about 2.32 units). Thus, SIWI improved students’ recount writing regardless of the fluency of students’ handwriting.

**Figure 2:**
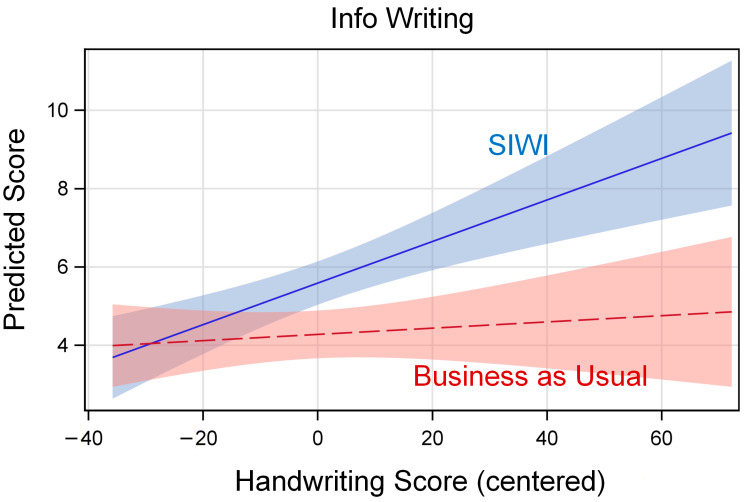
Moderation for Info Writing by Handwriting Fluency.

Figure 2 shows the relation between handwriting fluency and info writing. The lines are not parallel, with the SIWI line being substantially positive while the control line is nearly flat. The positive slope of the SIWI line suggests that students with higher levels of handwriting fluency were better able to benefit from the SIWI treatment during information report writing.

**Figure 3:**
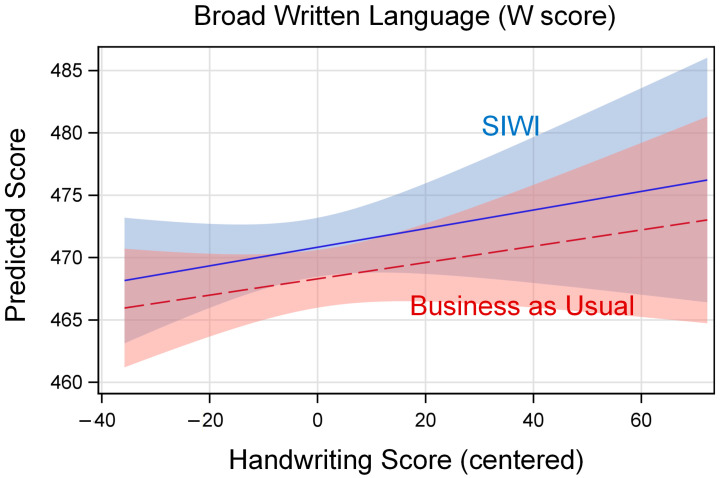
Moderation for Broad Written Language by Handwriting Fluency.

Figure 3 shows that there was a positive relation between handwriting fluency and broad written language W-score, but there was little difference between treatment and control, as evidenced by the high degree of overlap between the lines.

The slopes in Figure 1, Figure 2 and Figure 3 show a positive association between handwriting fluency and writing outcomes, indicating that students who write more fluently tend to perform better in writing tasks. However, the main effects of handwriting fluency were not statistically significant in the models. This suggests that, while students with more fluent handwriting tended to perform better as noted in the visual pattern, handwriting fluency did not independently predict outcomes once pretest scores and treatment conditions were accounted for.

## 4. Discussion

This nationwide study, involving 50 deaf education teachers and their 294 students randomly assigned to business-as-usual (BAU) and treatment groups, successfully replicates key findings from earlier experimental (K. Wolbers et al., 2015, 2020, 2022) and quasi-experimental studies (K. Wolbers, 2008, 2010; K. Wolbers et al., 2018, 2024). Consistent with prior research, SIWI demonstrated a substantial positive impact on genre-based outcomes for recount and information report writing among students in grades 3–6. Additionally, SIWI produced moderately large improvements in written language clarity within the recount genre and significant gains in motivation across both genres.

Another noteworthy finding was the small effect observed in favor of the treatment group on the WJ-IV broad written language composite measure, which was not statistically significant. This result contrasts with a prior study (K. Wolbers et al., 2022) where significant gains were documented, and the effect size was more substantial (i.e., 1.88 in the previous study compared to 0.27 here). One possible explanation is that the previous study, due to its small sample and non-equivalence at baseline, produced spurious results. The smaller effect size in this study may suggest that the measure is more distal to the writing skills targeted by other measures, which produced larger effect sizes. Another explanation is that the intervention’s impact on standardized language-related indices like the WJ-IV broad written language may not have been fully realized in this study due to the shortened school year when the treatment group reached its largest size. Depending on the school, instruction during the pandemic impacted 2.5 to 4 months of instruction. Even so, an effect size exceeding 0.25 is practically important, as recognized by the What Works Clearinghouse (see Graham et al., 2012).

These findings help clarify how SIWI produces strong instructional effects, which are situated within the writing instruction literature more broadly. The effects observed for genre-based writing outcomes are consistent with evidence showing that explicit, genre-focused instruction supports students’ ability to generate, organize, and elaborate ideas in text, and that interactive, collaborative writing provides the guidance and scaffolding students need for applying new writing skills (De Smedt & Van Keer, 2018; Englert et al., 2006; Graham et al., 2012; Graham & Perin, 2007). SIWI operationalizes these well-established instructional approaches in ways that are linguistically responsive to deaf learners (K. Wolbers et al., 2023c), particularly through co-construction of text, explicit attention to purpose and audience, and strategic mediation of the writing process. The consistency of effects across recount and information report writing in this replication strengthens confidence that SIWI supports core dimensions of writing through instructional approaches that are broadly effective while being deliberately designed to meet the linguistic strengths of deaf learners.

The pattern of language outcomes further aligns with prior writing research, indicating that language growth is most readily observed when assessed within meaningful composing contexts rather than through decontextualized measures (Graham et al., 2014; McCutchen, 1996). The moderate gains in grammatical clarity, as measured by the WER, observed in recount writing, suggest that SIWI supports students’ functional use of written language when linguistic instruction is embedded within authentic meaning-making activities and explicitly mediated to support language access and cross-language connections (K. Wolbers et al., 2023c). These findings are consistent with sociocognitive models of writing that emphasize the integration of language, content, and process (Applebee, 2000; Flower & Hayes, 1980; Hayes, 1996, 2006). In contrast, the smaller effects observed on the WJ-IV Broad Written Language suggest that the standardized composite assessment may reflect broad language abilities that are less tightly coupled to the genre-specific communicative tasks (c.f., Applebee & Langer, 2011; Schleppegrell, 2004) and may be particularly sensitive to reductions in instructional exposure, which disproportionately affected the SIWI cohort during COVID-related disruptions. These results suggest that SIWI contributes to strengthening students’ abilities to use language strategically in writing, particularly in genres where meaning, structure, and linguistic form are developed simultaneously through supportive interaction.

While many of the previously observed impacts on writing and language were effectively replicated, this study also revealed new insights into areas not extensively explored in earlier investigations. For instance, handwriting fluency emerged as a critical factor associated with stronger writing performance. A moderation analysis for information report writing identified an interaction effect: students with greater handwriting fluency appeared better equipped to benefit from SIWI in this genre. This finding suggests that fluency may enable students to focus more on composing content rather than grappling with the mechanics of writing—an advantage particularly relevant for less personalized genres like information reports.

Overall, these findings reaffirm the effectiveness of SIWI in improving deaf students’ writing quality and language clarity while underscoring the importance of tailored writing instruction that builds upon deaf learners’ unique linguistic and literacy strengths. The following sections will delve deeper into two specific findings—handwriting fluency and motivation—to explore how these factors contribute to students’ writing development within a linguistically responsive instructional framework.

### 4.1. Impact of Handwriting

Handwriting provides valuable insights into students’ writing knowledge and development. When transcription processes, such as handwriting, become sufficiently automatic, cognitive resources are freed to focus on higher-level tasks like composing, planning, and engaging in genre-specific thinking. This concept aligns with findings from reading fluency research (e.g., Duke & Cartwright, 2021; LaBerge & Samuels, 1974; Logan, 1997; Kuhn et al., 2010; Samuels, 2004, 2006), which suggest that automaticity in foundational skills supports advanced cognitive processes. Conversely, for writers with less fluent handwriting, transcription can remain effortful and consume working memory resources that would otherwise be allocated to critical processes such as idea generation, organization, and revision (McCutchen, 1996; Whitaker et al., 1994; Berninger, 1999).

Our results align with this framing within the context of linguistically responsive writing instruction for deaf elementary students. Specifically, handwriting fluency was positively related to recount writing across both SIWI and BAU groups, suggesting that students with greater fluency produced more robust recounts regardless of instructional condition. This underscores handwriting as a foundational skill for written expression. Additionally, a significant interaction emerged for information writing, indicating that students with higher handwriting fluency benefit more from SIWI than those with lower fluency. This pattern suggests that handwriting fluency may play a more critical role when genre demands increase. Informational texts require greater conceptual organization and domain-specific language (Schleppegrell, 2004), and when transcription is automatic, students can devote more cognitive resources to those higher-level composing tasks. Conversely, when transcription remains effortful, students may struggle to fully engage with the cognitive demands of genre-based instruction.

The positive association between handwriting fluency and writing outcomes replicates and extends prior findings. For example, Skar et al. (2022) found that handwriting fluency accounted for 7.4% of the variance in writing quality among primary students, after controlling for language background and other factors. Similarly, meta-analytic evidence indicates that handwriting instruction improves writing fluency and quality (López-Escribano et al., 2022). Together, these findings reinforce models of writing that indicate that automatic transcription plays a role in freeing cognitive resources for higher-order composing processes (Graham & Harris, 2000).

From an instructional perspective, these findings point to the need for a dual focus: developing composing skills through genre-based instruction and strengthening transcription skills, based in part on orthographic patterning. For deaf students, this includes explicit connections between handwriting and fingerspelling processes to support their unique linguistic needs (Haptonstall-Nykaza & Schick, 2007). Previous findings from research support that explicit attention to handwriting improves both fluency and overall writing quality (Santangelo & Graham, 2016) and that transcription skills remain important into the upper elementary grades (Graham et al., 1997).

### 4.2. Impact on Motivation

Across both recount and information genres, students who received SIWI reported higher motivation to write. This finding is noteworthy given that writing instruction for many deaf students has not always included consistent opportunities for genre-based writing that meaningfully supports engagement (K. Wolbers et al., 2019). Within SIWI, writing is framed as a communicative, collaborative, and purposeful process, supporting students in developing a sense of authorship and agency. These results mirror earlier findings in which student motivation increased across genres (K. Wolbers et al., 2022), and students engaged in SIWI began to self-identify as authors who wrote to communicate ideas and experiences (H. Dostal et al., 2015).

The theoretical model underlying SIWI integrates cognitive (Applebee, 2000; Flower & Hayes, 1980; Hayes, 1996, 2006) and sociocultural perspectives (Lave & Wenger, 2003; Rogoff, 1990; L. S. Vygotsky, 1978; L. Vygotsky, 1994; Wertsch, 1991), emphasizing that motivation develops through purposeful participation and collaboration within writing communities (De Smedt & Van Keer, 2014; Troia & Graham, 2002; K. Wolbers et al., 2022). Writing is viewed as both a strategic and goal-directed activity and a shared practice through which meaning is co-constructed. The SIWI framework sustains motivation by situating writing within authentic contexts, consistent with evidence that opportunities for strategic thinking, feedback, and interaction enhance engagement in writing (Camacho et al., 2020; Magnifico, 2010).

The gains in motivation in this study also align with research linking writing self-efficacy, persistence, and achievement (Graham & Harris, 2000; Pajares et al., 2007). As students participate in guided, collaborative writing and see their contributions in co-constructed texts, they develop stronger beliefs in their ability to communicate effectively through writing. Much like well-documented relationships between reading attitude and reading engagement (Guthrie & Wigfield, 2000), students who experience positive feelings towards writing are more likely to engage in writing tasks, which can strengthen their self-efficacy and reinforce continued participation and growth (Bruning & Horn, 2000; Pajares, 2003). This cyclical relationship affirms motivation as both an outcome and driver of literacy learning. Motivation, therefore, is not a peripheral affective outcome but a central mechanism through which writing knowledge, linguistic competence, and engagement are strengthened. Findings from this large-scale national study indicate that the design of SIWI, integrating shared authorship and opportunities for ongoing apprenticeship, metalinguistic reflection, and communicative purpose, supports not only growth in writing skills but also meaningful gains in motivation. SIWI appears to foster a reinforcing cycle in which increased motivation leads to greater engagement and, in turn, deeper skill development.

### 4.3. Limitations

As with many large-scale educational studies, this research faced contextual and logistical constraints that may have influenced findings. Most notably, the shortened school year and adjustments to data collection procedures during the COVID-19 pandemic reduced instructional exposure for the largest treatment cohort. This disruption may have constrained the extent to which language- and grammar-related effects were observable, particularly on standardized measures such as the WJ-IV. Additionally, while handwriting fluency emerged as an important moderator, it was measured through a timed transcription task that may not fully capture the nuanced relationships among fine-motor skill, orthographic processing, and linguistic encoding.

We acknowledge that background characteristics such as language proficiency can influence learning outcomes in deaf education (K. Wolbers et al., 2025); however, in this sample, there were no substantial differences in background variables across groups, minimizing concern about differential bias.

The study was conducted over an academic year, which may not have been sufficient to capture the full trajectory of language and writing growth that SIWI is designed to promote. Some effects may require extended exposure and cumulative practice to emerge. Additionally, the writing rubrics used in this study, while reliable and genre-specific, may have introduced ceiling or floor effects for students at the highest and lowest performance levels. For example, students with limited initial language access and/or production may have shown meaningful growth that fell outside rubric thresholds.

### 4.4. Future Directions

Future research could continue to examine how the SIWI framework can be applied across a broad range of writing contexts, including additional grade levels, genres, and instructional settings. Across randomized controlled trials, quasi-experimental studies, and qualitative studies, SIWI has consistently demonstrated positive effects on deaf students’ writing, language, and motivation. Expanding this work could further show how SIWI scaffolds students’ movement from narrative writing toward disciplinary genres that require abstract reasoning and specialized vocabulary.

This study advances understanding of how SIWI supports writing development and opens opportunities to further examine the instructional and linguistic processes underlying its effects. One process involves the coordination of transcription fluency, linguistic knowledge, and higher-order composing. Although handwriting fluency was positively associated with writing performance, its influence differed by genre. In information writing, students with greater fluency benefited more from SIWI. This pattern suggests that SIWI’s emphasis on interactive meaning-making and metalinguistic engagement may help students manage transcription-level constraints, while students with greater fluency are positioned to take fuller advantage of those supports in more complex genres. These findings point to the value of examining how specific linguistic and cognitive processes interact within SIWI to shape writing development over time.

Relatedly, the role of fingerspelling and orthographic knowledge in supporting handwriting fluency is another area for future research. An awareness of letter-sequence patterns may support short-term memory for efficient encoding. It remains unclear what the exact role is and to what extent evidence-based fingerspelling or word-pattern instruction promotes automatic word- and phrase-level processing among deaf students. If these foundational components are compromised, students may rely on letter-by-letter transcription, limiting fluency. Future work could explore how these interrelated domains contribute to fluent written expression and how SIWI might further leverage these relationships.

## 5. Conclusions

This randomized controlled trial strengthens the evidence base for SIWI by replicating and extending prior findings of its positive effects on deaf students’ writing quality, language clarity, and motivation across diverse contexts. As a replication study, it reaffirms the reliability and generalizability of SIWI’s outcomes while contributing new insights into the role of handwriting fluency in writing development. Results from this and previous investigations confirm that instructional frameworks emphasizing interactive meaning-making foster both skill growth and engagement. Furthermore, these findings underscore the importance of writing instruction that leverages deaf students’ linguistic resources and promotes purposeful communication. As research continues to investigate the intricate relationship between language development and literacy among deaf students, SIWI stands out as a robust model for genre-based instruction and professional learning initiatives that promote equity and access in writing education.

## Figures and Tables

**Table 1:** Teacher Demographics (*n* = 50).

Variable	Value	Treatment	BAU	Total	%
Gender	Female	26	23	49	98
	Male	0	1	1	2
Ethnicity/race	White	24	23	47	94
	African American	1	1	2	4
	Asian/Pacific Islander	1		1	2
Hearing status	Deaf	4	4	8	16
	Hearing	22	20	42	84
Degree	Advanced Graduate	2	4	6	12
	Graduate	19	17	36	72
	Undergraduate	5	3	8	16
	Total	26	24	50	100

**Table 2:** Student Demographics (*n* = 294).

Variable	Value	Treatment	BAU	Total	%
Gender	Female	63	63	126	43
	Male	101	65	166	56
	Not reported	2	0	2	1
Ethnicity/race	White	71	49	120	41
	African American	40	29	69	23
	Latine	28	31	59	20
	Asian/Pacific Islander	13	6	19	6
	Native American	1	0	1	1
	Multiracial	8	7	15	5
	Other	5	6	11	4
Grade	Not reported	0	1	1	1
	3	25	24	49	17
	4	43	29	72	24
	5	52	29	81	27
	6	46	45	91	31
Disability	Not reported	2	0	2	1
	No additional disability	127	99	226	77
	Additional disability	37	29	66	22
	Total	166	128	294	100

**Table 3:** Ratings of Students’ Proficiency in ASL, Spoken English, and Language on a Scale of 1–5 (*n* = 294).

	ASL, *n* (%)		Spoken English, *n* (%)		Language *^a^*, *n* (%)	
	Treatment	BAU	Treatment	BAU	Treatment	BAU
Can express most anything (1)	43 (26%)	32 (25%)	38 (23%)	21 (16%)	71 (44%)	44 (34%)
Can express many things (2)	46 (28%)	37 (29%)	35 (21%)	31 (24%)	55 (33%)	50 (39%)
Difficulty expressing many things (3)	34 (21%)	27 (21%)	25 (15%)	23 (18%)	29 (18%)	23 (18%)
Difficulty expressing most things (4)	11 (7%)	6 (5%)	9 (5%)	14 (11%)	7 (4%)	8 (6%)
Does not use the language (5)	28 (17%)	25 (20%)	55 (33%)	38 (30%)	–	–
Not reported	4 (2%)	1 (1%)	4 (2%)	1 (1%)	4 (2%)	1 (1%)

Note. Percentages are rounded and may not total exactly 100%. *^a^* “Language” refers to students’ highest proficiency level in ASL and/or spoken English.

**Table 4:** Pre- and Post-Intervention Descriptive Statistics for Writing, Language, Motivation and Handwriting Fluency Outcomes by Treatment Group.

Outcome	Wave	Group	*n*	Mean	SD	Min	Max
Recount Writing Total	Pre	BAU	128	4.25	2.96	0	13
	Pre	Treatment	166	3.88	2.87	0	14
	Post	BAU	107	4.49	3.12	0	14
	Post	Treatment	123	6.50	3.70	0	15
Info Writing Total	Pre	BAU	128	3.84	3.07	0	14
	Pre	Treatment	164	3.43	2.54	0	12
	Post	BAU	103	4.47	3.14	0	18
	Post	Treatment	122	5.53	3.59	0	15
Recount WER III	Pre	BAU	84	0.69	0.27	0	1
	Pre	Treatment	109	0.71	0.26	0	1
	Post	BAU	80	0.68	0.28	0	1
	Post	Treatment	94	0.74	0.26	0	1
Info WER III	Pre	BAU	86	0.73	0.26	0	1
	Pre	Treatment	94	0.70	0.29	0	1
	Post	BAU	72	0.73	0.24	0	1
	Post	Treatment	88	0.71	0.25	0	1
Broad Written Language	Pre	BAU	126	461.07	26.78	377	509
	Pre	Treatment	162	461.89	26.10	379	516
	Post	BAU	95	467.21	26.28	361	521
	Post	Treatment	91	471.97	21.11	392	513
Recount Motivation	Pre	BAU	88	3.88	0.83	2.27	5.93
	Pre	Treatment	124	4.02	1.01	0.47	5.73
	Post	BAU	78	4.01	0.84	2.00	6.00
	Post	Treatment	78	4.29	0.81	1.07	5.80
Info Motivation	Pre	BAU	101	3.86	0.83	1.60	5.60
	Pre	Treatment	124	4.11	0.93	1.47	6.00
	Post	BAU	75	3.96	0.94	1.80	6.00
	Post	Treatment	78	4.41	0.82	2.11	6.00
Handwriting Fluency	Pre	BAU	128	37.54	19.56	1.33	109.33
	Pre	Treatment	166	36.80	18.76	2.00	94.67
	Post	BAU	103	41.67	19.58	7.33	84.67
	Post	Treatment	112	43.45	21.42	6.67	97.33

**Table 5:** Baseline Differences for Treatment versus BAU (Cohen’s *d*).

Pretest	*d*
Recount Writing	−0.13
Info Writing	−0.15
Recount WER III	0.08
Info WER III	−0.11
Broad Written Language	0.03
Recount Motivation	0.15
Info Motivation	0.28
Handwriting Fluency	−0.04

**Table 6:** Multilevel Model Estimates of Group Treatment Effects Across Measured Outcomes.

Outcome	Fixed Effects	Est.	SE	*df*	*t*	*p*
Recount Writing	Intercept	4.46	0.30	45	15.11	<0.01
	Classroom pretest	1.07	0.09	60	11.26	<0.01
	Child pretest	0.63	0.11	162	5.64	<0.01
	SIWI	2.37	0.40	45	5.87	<0.01
	Pretest * SIWI	0.06	0.16	169	0.37	0.71
	Effect size *(g)*	*1.11*				
	**Random Effects**	Est.	SD			
	Classroom	1.09	1.04			
	Residual	4.55	2.13			
Info Writing	**Fixed Effects**	Est.	SE	*df*	*t*	*p*
	Intercept	4.38	0.32	50	13.43	<0.01
	Classroom pretest	0.84	0.10	64	8.08	<0.01
	Child pretest	0.78	0.10	160	7.58	<0.01
	SIWI	1.38	0.44	50	3.10	<0.01
	Pretest * SIWI	0.10	0.14	160	0.74	0.46
	*Effect size (g)*	*0.72*				
	**Random Effects**	Est.	SD			
	Classroom	1.77	1.33			
	Residual	3.72	1.93			
Recount WER III	**Fixed Effects**	Est.	SE	*df*	*t*	*p*
	Intercept	0.70	0.03	37	26.29	<0.01
	Classroom pretest	0.79	0.08	49	9.42	<0.01
	Child pretest	0.85	0.13	84	6.55	<0.01
	SIWI	0.09	0.04	36	2.53	0.02
	Pretest * SIWI	−0.42	0.17	88	−2.50	0.01
	*Effect size (g)*	*0.64*				
	**Random Effects**	Est.	SD			
	Classroom	0.01	0.08			
	Residual	0.02	0.14			
Info WER III	**Fixed Effects**	Est.	SE	*df*	*t*	*p*
	Intercept	0.76	0.02	36	38.23	<0.01
	Classroom pretest	0.70	0.06	70	10.68	<0.01
	Child pretest	0.53	0.09	86	5.90	<0.01
	SIWI	0.03	0.03	40	0.92	0.36
	Pretest * SIWI	−0.38	0.13	89	−2.88	<0.01
	*Effect size (g)*	*0.20*				
	**Random Effects**	Est.	SD			
	Classroom	0.00	0.04			
	Residual	0.02	0.13			
Broad Written Language	**Fixed Effects**	Est.	SE	*df*	*t*	*p*
	Intercept	468.92	1.16	53	406.50	<0.01
	Classroom pretest	0.93	0.04	59	23.07	<0.01
	Child pretest	0.83	0.06	146	14.88	<0.01
	SIWI	2.37	1.65	53	1.46	0.15
	Pretest * SIWI	−0.16	0.09	152	−1.82	0.07
	*Effect size (g)*	*0.27*				
	**Random Effects**	Est.	SD			
	Classroom	11.23	3.35			
	Residual	76.96	8.77			
Recount Motivation	**Fixed Effects**	Est.	SE	*df*	*t*	*p*
	Intercept	3.94	0.10	42	39.93	<0.01
	Classroom pretest	0.52	0.11	52	4.52	<0.01
	Child pretest	0.46	0.12	99	3.86	<0.01
	SIWI	0.31	0.14	44	2.26	0.03
	Pretest * SIWI	−0.21	0.16	106	−1.30	0.20
	*Effect size (g)*	*0.49*				
	**Random Effects**	Est.	SD			
	Classroom	0.08	0.29			
	Residual	0.40	0.63			
Info Motivation	**Fixed Effects**	Est.	SE	*df*	*t*	*p*
	Intercept	3.95	0.10	39	38.66	<0.01
	Classroom pretest	0.56	0.12	65	4.52	<0.01
	Child pretest	0.42	0.13	105	3.20	<0.01
	SIWI	0.36	0.14	44	2.49	0.02
	Pretest * SIWI	−0.19	0.19	107	−0.99	0.32
	*Effect size (g)*	*0.47*				
	**Random Effects**	Est.	SD			
	Classroom	0.05	0.23			
	Residual	0.58	0.76			

Note: Est. = Estimate. SE = standard error. SD = standard deviation. Effect size is the model-based intervention difference (Hedges, 2007).

**Table 7:** Moderation Analyses.

Outcome	Fixed Effects	Est.	SE	*df*	*t*	*p*
Recount Writing	Intercept	4.46	0.29	41	15.36	<0.01
	Classroom pretest	0.96	0.10	74	9.27	<0.01
	Child pretest	0.55	0.12	179	4.46	<0.01
	SIWI	2.32	0.40	41	5.84	<0.01
	Pretest * SIWI	0.07	0.17	182	0.42	0.67
	Handwriting	0.02	0.01	219	1.46	0.14
	Handwriting * SIWI	0.01	0.02	195	0.53	0.59
	**Random Effects**	Est.	SD			
	Classroom	1.03	1.01			
	Residual	4.49	2.12			
Info Writing	**Fixed Effects**	Est.	SE	*df*	*t*	*p*
	Intercept	4.40	0.31	51	14.22	<0.01
	Classroom pretest	0.75	0.10	78	7.11	<0.01
	Child pretest	0.75	0.11	174	6.64	<0.01
	SIWI	1.31	0.42	50	3.09	<0.01
	Pretest * SIWI	0.04	0.14	173	0.27	0.78
	Handwriting	0.01	0.01	215	0.63	0.53
	Handwriting * SIWI	0.04	0.02	215	2.60	0.01
	**Random Effects**	Est.	SD			
	Classroom	1.55	1.25			
	Residual	3.50	1.87			
Broad Written Language	**Fixed Effects**	Est.	SE	*df*	*t*	*p*
	Intercept	468.81	1.18	51	396.80	<0.01
	Classroom pretest	0.89	0.05	76	19.17	<0.01
	Child pretest	0.80	0.06	155	12.84	<0.01
	SIWI	2.59	1.69	51	1.53	0.13
	Pretest * SIWI	−0.14	0.09	157	−1.58	0.12
	Handwriting	0.06	0.06	177	1.14	0.26
	Handwriting * SIWI	0.01	0.08	158	0.11	0.91
	**Random Effects**	Est.	SD			
	Classroom	12.93	3.60			
	Residual	75.75	8.70			

## Data Availability

The data will be publicly available at this DOI (https://doi.org/10.5281/zenodo.16420315 accessible on 24 December 2025) following completion of the project’s final analyses.

## Note

1 We calculated minimal detectable effect sizes in Optimal Design using information from our previous studies: 80% power, alpha = 0.05, 4 students per classroom, ICC = 30%, and pretest R-squared of 36%. For a range of participating teachers from 46 to 67, we found a range of MDES of 0.36 to 0.60.

## Appendix A

**Table A1:** Variance Components and ICC for All Outcomes.

Outcome	Wave	Classroom	Student	ICC
Recount Writing	pretest	3.67	5.00	0.42
	posttest	6.90	6.27	0.52
Info Writing	pretest	2.72	5.01	0.35
	posttest	4.38	6.99	0.39
Recount WER III	pretest	0.04	0.04	0.49
	posttest	0.03	0.04	0.43
Info WER III	pretest	0.02	0.05	0.31
	posttest	0.03	0.03	0.49
WJ Broad Writing	pretest	347.46	353.61	0.50
	posttest	315.19	258.86	0.55
Recount Motivation	pretest	0.04	0.85	0.04
	posttest	0.20	0.50	0.28
Info Motivation	pretest	0.09	0.71	0.12
	posttest	0.15	0.68	0.18
Handwriting Fluency	pretest	99.32	265.50	0.27
	posttest	174.40	239.57	0.42

Note. ICC = intraclass correlation, or the ratio of classroom to total variance.

**Table A2:** FIML estimates of effect sizes (Hedges *g*).

Outcome	Est	SE	Ratio	*p*
Recount Writing	1.09	0.21	5.09	<0.01
Info Writing	0.73	0.20	3.58	<0.01
Recount WER III	0.45	0.19	2.39	0.02
Info WER III	0.10	0.19	0.50	0.62
WJ Broad Writing	0.24	0.17	1.42	0.16
Recount Motivation	0.39	0.22	1.76	0.08
Info Motivation	0.46	0.17	2.68	0.01

Note. These are effect sizes calculated as the model-predicted treatment difference divided by the residual SD (Hedges, 2007). The model included pretest and posttest nested within classrooms with FIML estimation assuming missing at random. Effect sizes here can be compared to those reported in Table 5.
